# Berkeley Screen: a set of 96 solutions for general macromolecular crystallization

**DOI:** 10.1107/S1600576717011347

**Published:** 2017-09-05

**Authors:** Jose H. Pereira, Ryan P. McAndrew, Giovani P. Tomaleri, Paul D. Adams

**Affiliations:** a Molecular Biophysics and Integrated Bioimaging Division, Lawrence Berkeley National Laboratory, Berkeley, CA 94720, USA; b Joint BioEnergy Institute, Emeryville, CA 94608, USA; c Department of Bioengineering, University of California, Berkeley, CA 94720, USA

**Keywords:** crystallization screens, crystal packing, ions and buffers, crystal growth, structural biology

## Abstract

Using statistical analysis of the Biological Macromolecular Crystallization Database, combined with previous knowledge about crystallization reagents, a crystallization screen called the Berkeley Screen has been created. Correlating crystallization conditions and high-resolution protein structures, it is possible to better understand the influence that a particular solution has on protein crystal formation. Ions and small molecules such as buffers and precipitants used in crystallization experiments were identified in electron density maps, highlighting the role of these chemicals in protein crystal packing. The Berkeley Screen has been extensively used to crystallize target proteins from the Joint BioEnergy Institute and the Collaborative Crystallography program at the Berkeley Center for Structural Biology, contributing to several Protein Data Bank entries and related publications. The Berkeley Screen provides the crystallographic community with an efficient set of solutions for general macromolecular crystallization trials, offering a valuable alternative to the existing commercially available screens.

## Abstract 2

The Berkeley Screen provides an efficient set of solutions for general macromolecular crystallization trials.

## 1. Introduction   

X-ray crystallography is currently the most successful technique used to solve macromolecular structures, contributing several thousand new entries to the Protein Data Bank (PDB) every year (Berman *et al.*, 2000 ▸). Recently the PDB reached 125 000 structure entries, with approximately 90% of these entries coming from X-ray crystallography, 10% from nuclear magnetic resonance (NMR) and 1% from electron microscopy. While the number of new entries using X-ray crystallography increases every year, the number of new NMR entries has been decreasing since 2007. In contrast, new entries using cryo-electron microscopy (cryo-EM) are increasing rapidly as a result of recent technical advances enabling near atomic resolution data collection (Zhou, 2008 ▸; Baker *et al.*, 2010 ▸). In 2016, cryo-EM was responsible for 410 new entries in the PDB, almost equal to the 453 new entries from NMR, suggesting that cryo-EM will soon overtake the latter technique for solving macromolecular structures (PDB, 2017 ▸).

There are three important stages in going from the purified sample of a macromolecule(s) to a description of the structure in a scientific paper: observation of the crystal in the microscope, obtaining the first X-ray diffraction pattern, and achieving an electron density map of sufficient quality that the structure can be solved. The crystal is the critical starting point for X-ray data collection, and consequently, its properties are correlated with the quality of the electron density maps and the level of detail that can be extracted for a macromolecular structure. Currently, a complete X-ray data set often can be obtained using synchrotron storage ring sources equipped with a pixel array detector such as the Pilatus in a matter of minutes. Crystallographic packages such as *Phenix* (Adams *et al.*, 2010 ▸; Afonine *et al.*, 2012 ▸) and the *CCP4 Suite* (Winn *et al.*, 2011 ▸) provide the structural biology community with very powerful tools for solving and completing crystal structures. However, the crystallization step still is less developed compared to X-ray data collection and structure solution.

Despite the fact that robots make it possible to efficiently screen against hundreds of crystallization solutions, crystallization is still a trial and error process, and many attempts still fail. Moreover, even after a crystal with good shape and size has been obtained, there is no guarantee that the crystal will diffract well. Therefore, understanding the agents and factors involved in the crystallization process could be crucial to obtaining a macromolecular structure. In this paper we describe a set of 96 solutions to be used for general macromolecular crystallization trials. Furthermore, we have correlated the solvent content of a set of high-resolution crystal structures to explain crystal growth in one particular solution.

## 2. Results and discussion   

### 2.1. Designing the Berkeley Screen formulation   

The development of the new protein screen started with the observation that a significant number of macromolecular structures contain bound cation/anion or small molecule ligands from the crystallization solution. In addition, the low success rate of some commercially available protein crystallization screens motived the creation of a new screen in order to optimize cost and time.

In some cases, a metal ion is important for protein activity, such as the Mg^2+^ ion that is found coordinating the phosphate groups in ATP binding proteins (Pereira, Ralston *et al.*, 2010 ▸, 2012 ▸). Although these catalytic site metals are unlikely to be involved in crystal packing interactions, they are often important in stabilizing a region of the macromolecule and therefore have an impact on the crystallization process. In other cases, the metal ion or small molecules, such as buffer components, play an important role in creating crystal contacts between the macromolecules in the lattice. Using the ions that are commonly bound to biological macromolecular structures (Mg^2+^, Ca^2+^, K^+^, Na^+^, Cl^1−^, SO_4_^2−^, PO_4_^2−^) combined with the most successful crystallization reagents (Tung & Gallagher, 2009 ▸) and a revised set of crystallization reagents (McPherson & Gavira, 2014 ▸; Bergfors, 2008 ▸; McPherson, 2001 ▸, 1976 ▸; Ames *et al.*, 1998 ▸; Trakhanov & Quiocho, 1995 ▸; Jancarik & Kim, 1991 ▸), we created a sparse-matrix screen of 96 crystallization solutions (supplementary file S1). The occurrences of the most common salts, precipitants and pH ranges in the Biological Macromolecular Crystallization Database (BMCD; Tung & Gallagher, 2009 ▸) and Berkeley Screen solutions are described in Table 1 ▸.

**Figure 1:**
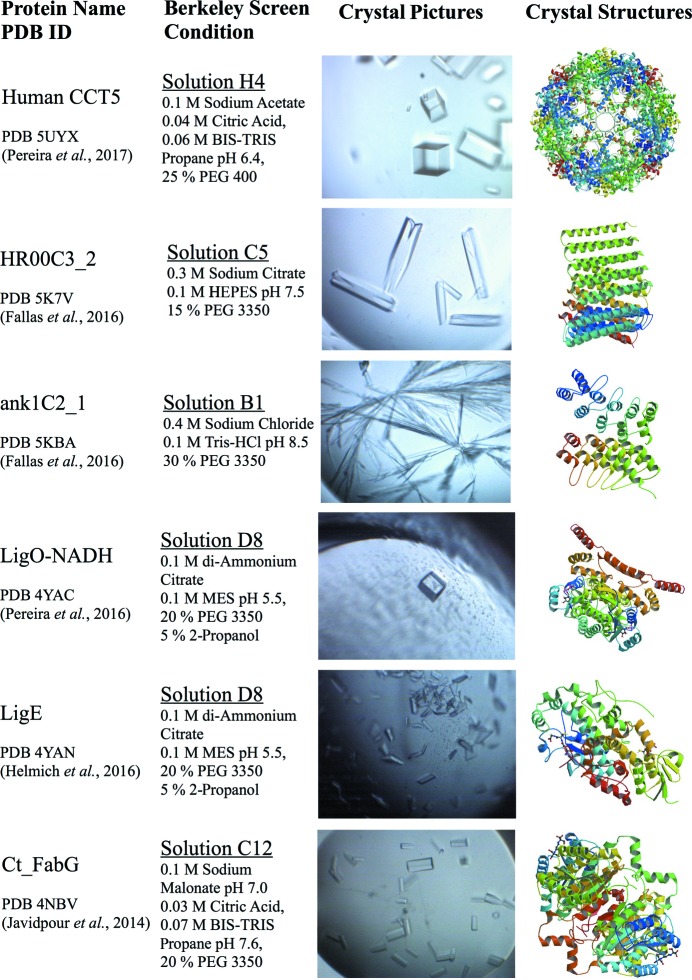
Examples of protein crystals successfully grown from Berkeley Screen solutions containing PEGs, which have the highest occurrence in the Berkeley Screen formulation. The human TRiC subunit CCT5 (PDB code 5uyx) crystallized in PEG 400 as precipitant, whereas the computational design proteins HR00C3_2 (PDB code 5k7v) and ank1C2_1 (PDB code 5kba), the lignin degradation enzymes LigO (PDB code 4yac) and LigE (PDB code 4yan), and a reductase involved in fatty acid biosynthesis Ct_FabG (PDB code 4nbv) crystallized in PEG 3350.

In 1968, an alcohol oxidase was the first protein crystallized using a polyethylene glycol (PEG) (Janssen & Ruelius, 1968 ▸; Kirkwood *et al.*, 2015 ▸). PEGs became extensively used for crystallization after the McPherson study using PEGs of various molecular weights was published in 1976 (McPherson, 1976 ▸). In addition, a study published by Newman and collaborators using a systematic pH, anion and cation testing (PACT) with the precipitants PEG 1500, PEG 3350 and PEG 6000 contributed to wider use of the PEGs for an initial rational protein crystallization screening (Newman *et al.*, 2005 ▸). PEGs compete with the protein for water interaction, forcing the protein out of solution and consequently promoting crystal nucleation and crystal growth. Currently, PEGs (including all molecular weights) are the most successful precipitants used in crystallization, responsible for about half of all entries in the BMCD (Tung & Gallagher, 2009 ▸). PEGs therefore have the highest occurrence in the Berkeley Screen as compared to any other reagent. In the formulation are 70 solutions (73%) containing PEGs, more specifically 36 solutions with PEG 3350, nine solutions with PEG 400, nine solutions with PEG monomethyl ether (MME) 2000, five solutions with PEG MME 5000, four solutions with PEG MME 550, three solutions with PEG 4000, two solutions with PEG 1500, one solution with PEG 8000 and one solution with PEG 10 000. The significant difference between the occurrence of PEG in the BMCD (∼50%) and in the Berkeley Screen (∼70%) is due to the use of PEGs in combination with other precipitants. For example, condition D8 (solution 45 of the Berkeley Screen) has two components as precipitants: 20% PEG 3350 and 5% 2-propanol. As expected from the high occurrence among the conditions present in the Berkeley Screen formulation, the solutions containing PEG as a precipitant are the most successful for the target proteins tested from the Joint BioEnergy Institute and the Collaborative Crystallography program at the Berkeley Center for Structural Biology (Pereira *et al.*, 2017 ▸, 2016 ▸; Fallas *et al.*, 2016 ▸; Mills *et al.*, 2016 ▸; Helmich *et al.*, 2016 ▸; Javidpour *et al.*, 2014 ▸) (Fig. 1 ▸).

A comparison of 12 different salts used in crystallization solutions, including ammonium sulfate, sodium malonate, lithium sulfate, ammonium phosphate, sodium phosphate, sodium citrate, sodium acetate, sodium tartrate, magnesium sulfate, sodium chloride, ammonium formate and lithium chloride, showed that sodium malonate was the most successful salt for the 31 proteins and virus targets tested (McPherson, 2001 ▸). Small organic acids such as citrate, tartrate, acetate, malonate and formate are valuable precipitants for crystal growth (McPherson, 2001 ▸). Following this study by McPherson, we created 11 solutions specifically containing sodium malonate in the Berkeley Screen formulation, varying the concentration from 0.1 to 2.2 *M* and the pH range from 5.0 to 8.0. Moreover, 36 solutions of the screen include small organic acids (supplementary file S1).

### 2.2. Understanding the influence of crystallization solutions on protein crystal formation   

We selected four protein structures solved recently in our laboratory at high resolution (1.1–1.8 Å) to illustrate how reagents found in the Berkeley Screen influenced crystal formation.

#### 2.2.1. Symmetrical ion coordination (magnesium – Mg^2+^)   

**Figure 2:**
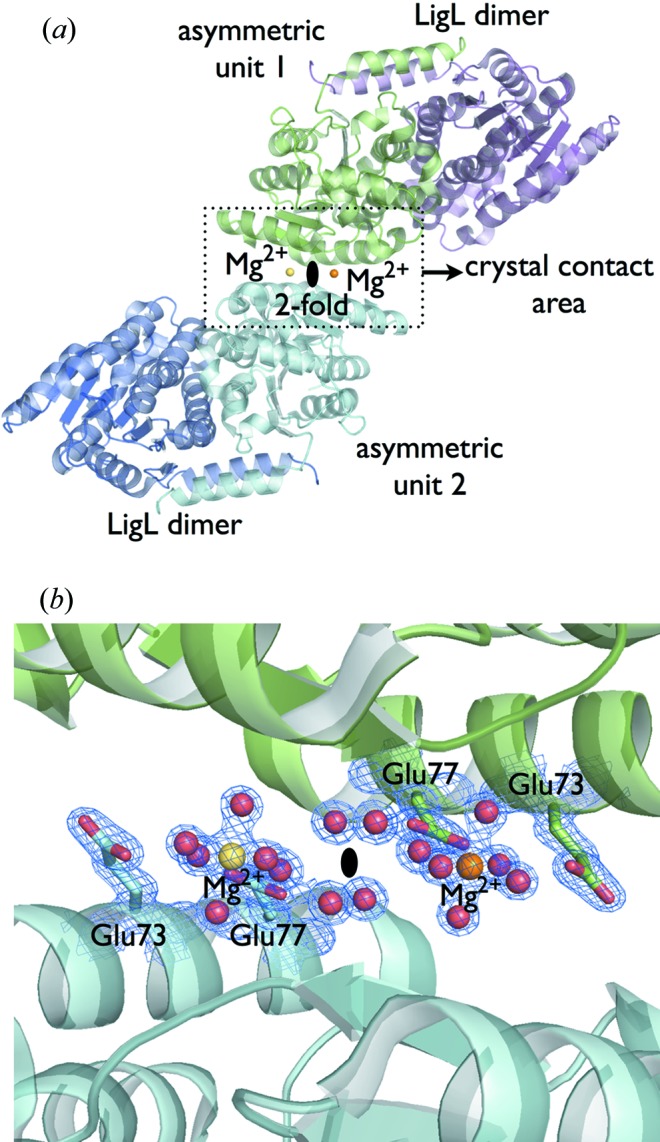
(*a*) The LigL structure (PDB code 4yai; Pereira *et al.*, 2016 ▸) was solved at 1.6 Å resolution with two molecules per asymmetric unit, which represents the biological dimer, showing Mg^2+^ was essential for the crystal lattice contacts. A twofold (*C*2) symmetry axis is shown with a solid oval symbol. (*b*) A 2*mF*_o_–*DF*_c_ electron density map for the LigL structure contoured at 1.5σ is shown in blue around the Mg^2+^ ions, the water molecules, and the side chains of Glu73 and Glu77. The octahedral coordination of Mg^2+^ is water mediated by the negative surface residues Glu73 and Glu77, generating the major crystal contact between symmetry-related molecules. Water molecules are shown as red spheres.

Free magnesium (Mg^2+^) is an essential cation for a broad range of enzymes. In mammalian cells, more than 350 enzymes require or are regulated by Mg^2+^ (Romani, 2013 ▸). Usually, Mg^2+^ is bound to the protein *via* negatively charged residues such as aspartate (Asp) or glutamate (Glu) that are involved in the common Mg^2+^ octahedral coordination (Lebbink *et al.*, 2010 ▸). However, the Mg^2+^ ion could also bind directly or through water coordination to negatively charged Asp and Glu residues that are exposed to solvent, potentially creating crystal contacts between molecules in the lattice. An example is the crystal formation of the NAD-dependent de­hydrogenase LigL from *Sphingobium sp.* strain (Pereira *et al.*, 2016 ▸). LigL was crystallized from 0.2 *M* magnesium chloride, 0.1 *M* HEPES pH 7.5 and 25% PEG 3350. The LigL structure was solved at 1.6 Å resolution, revealing that Mg^2+^ was essential for the crystal lattice contacts. The Mg^2+^ was water coordinated and held in place by surface negative residues Glu73 and Glu77, generating the major crystal contacts between symmetry mates (Fig. 2 ▸). Statistical analysis of the BMCD showed that 10% of all the entries had magnesium chloride present in the crystallization solution. Therefore, the Berkeley Screen was designed with 16 solutions (16%) containing magnesium chloride from 0.005 to 0.6 *M* concentration.

#### 2.2.2. The classic, ammonium sulfate – hydrogen-bond and salt bridge interactions   

**Figure 3:**
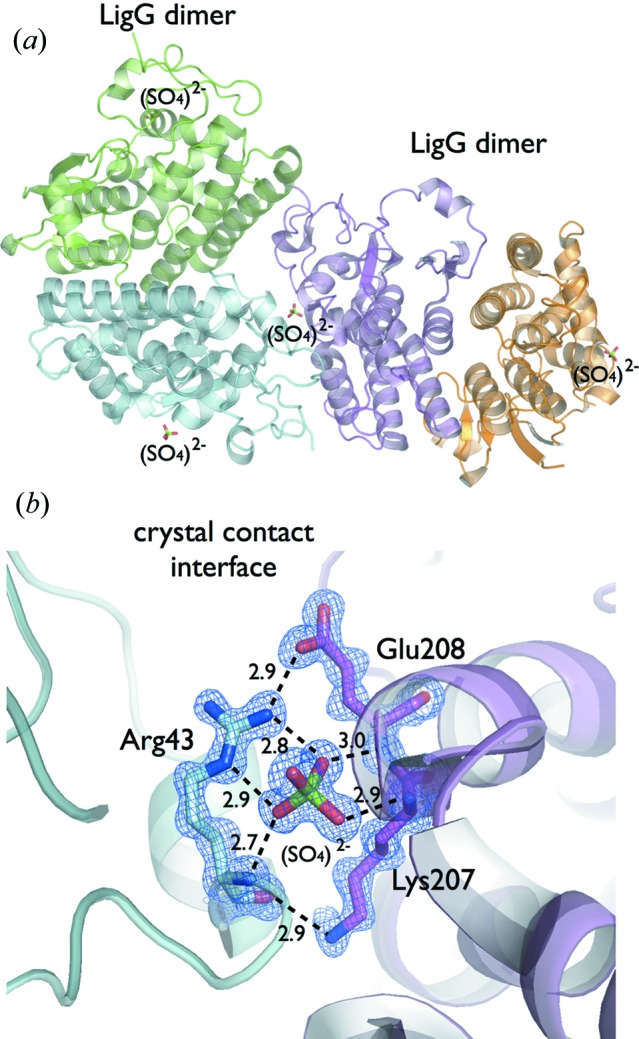
(*a*) Crystal packing in the dimeric crystal structure of a gluta­thione-dependent lyase LigG solved at 1.1 Å resolution (PDB code 4yap) (Pereira *et al.*, 2016 ▸), showing the SO_4_^2−^ anion bound to the surface region participating in the crystal lattice. (*b*) Zoom in showing the LigG crystal contact with the hydrogen bonds and salt bridges observed between SO_4_^2−^ and the symmetry-related copies of LigG. The SO_4_^2−^ contacts Arg43 *via* the main chain N atom and NE and NH_2_ from the side chain. The positive residue Arg43 makes a salt bridge interaction with the symmetry-related residue Glu208. The SO_4_^2−^ ion makes additional contacts with the main chain N atoms of Glu208 and Lys207. The NZ atom of Lys207 makes a hydrogen bond with the carbonyl group of Arg43. A 2*mF*_o_–*DF*_c_ electron density map contoured at 1.5σ is shown in blue. Contacts are shown as broken lines and distances in ångströms.

Historically, the most successful crystallization precipitant was ammonium sulfate. More recently, the most common precipitant has become PEG 3350 (Kirkwood *et al.*, 2015 ▸). However, analysis of the BMCD showed that ammonium sulfate is still a good precipitant candidate for crystallization trials, corresponding to 26% of all entries (Tung & Gallagher, 2009 ▸). Exploring the high-resolution crystal structure of a gluta­thione-dependent lyase LigG solved at 1.1 Å resolution (Pereira *et al.*, 2016 ▸), we identified a sulfate ion (SO_4_^2−^) participating in crystal contacts. The LigG was crystallized from 0.1 *M* bis-tris propane pH 7.0 and 1.5 *M* ammonium sulfate. The SO_4_^2−^ ion interacts with Arg43 *via* the N main chain and NE and NH_2_ atoms from the side chain. The positive residue Arg43 makes a salt bridge interaction with the LigG symmetry mate residue Glu208. The SO_4_^2−^ ion makes additional contacts with the main chain N atoms of Glu208 and Lys207. The NZ atom of Lys207 makes a hydrogen bond with the carbonyl group of Arg43 (Fig. 3 ▸). Therefore, SO_4_^2−^ plays a central role in stabilizing residue Arg43, creating a contact for the negatively charged residue Glu208 from a symmetry-related molecule. How the ammonium cation NH_4_^+^ participates in crystallization is challenging to determine owing to the difficulty of distinguishing free NH_4_^+^ and water molecules in the electron density map. However, it is known that NH_4_^+^ is the most robust cation for precipitation of hen egg white proteins from the Hofmeister series (Hofmeister, 1890 ▸; Bergfors, 2008 ▸). The NH_4_^+^ ions compete with the protein for water molecule interactions, forcing the protein out of solution: the salting-out effect. The capability of NH_4_^+^ to compete for water interactions, and the ability of SO_4_^2−^ to make hydrogen bonds or salt bridges between symmetry-related molecules, make ammonium sulfate salt a reagent that must be presented in an initial protein crystallization screen. The Berkeley Screen has 12 conditions containing ammonium sulfate.

## 3. Intermolecular interactions induced by cadmium – Cd^2+^   

**Figure 4:**
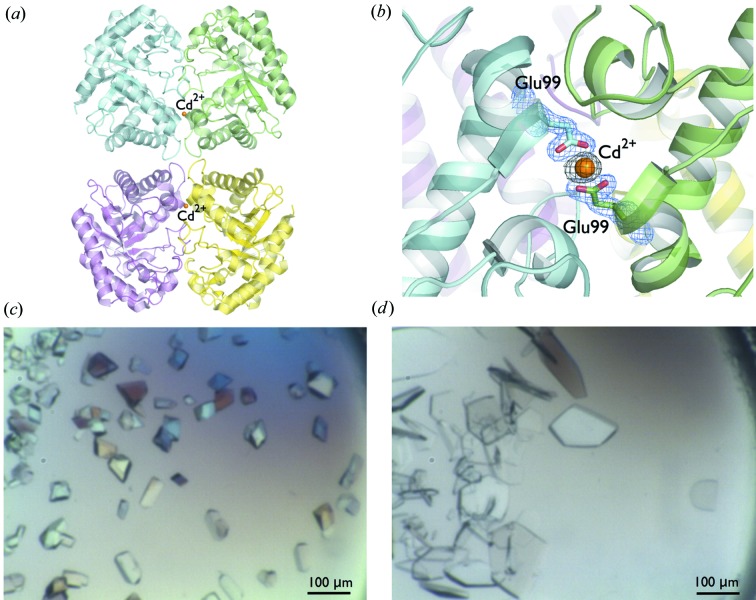
(*a*) The asymmetric unit contents showing four molecules of Cel5A (PDB code 3mmw; Pereira *et al.*, 2010 ▸). (*b*) The Cel5A structure showed that cadmium was essential to form ‘bridges’ across the interface of protein molecules through coordination by the negative charge residue Glu99 present at the surface of the protein. A 2*mF*_o_–*DF*_c_ electron density map contoured at 1.5σ is shown in blue around the Glu99 residues and an *mF*_o_–*DF*_c_ electron density map contoured at 10σ when the divalent metal was omitted from the model is shown in black around Cd^2+^. (*c*) The presence of Ni^2+^ and Cd^2+^ produced Cel5A crystals belonging to the *P*1 space group that were solved at 2.2 Å resolution. The crystallization condition of these crystals was 0.02 *M* nickel(II) chloride, 0.02 *M* magnesium chloride, 0.02 *M* cadmium chloride, 0.1 *M* sodium acetate trihydrate pH 4.5 and 16% polyethylene glycol monomethyl ether 2000. (*d*) When only Cd^2+^ was included in the crystallization solution, Cel5A crystals belonging to the *P*2_1_ space group were produced with a completely different crystal morphology, which were solved at 1.8 Å resolution. The crystallization condition for the Cel5A *P*2_1_ crystals was 0.02 *M* cadmium chloride, 0.1 *M* sodium acetate trihydrate pH 4.5 and 16% polyethylene glycol monomethyl ether 2000 (Pereira *et al.*, 2010 ▸).

Cadmium (Cd^2+^) ions can shield charges located at protein surface regions, creating favorable intermolecular interactions (Trakhanov & Quiocho, 1995 ▸; Ames *et al.*, 1998 ▸). Analysis of a glycoside hydrolase Cel5A structure (Pereira, Chen *et al.*, 2010 ▸) revealed that cadmium was essential in forming ‘bridges’ between protein molecules involving the negatively charged residue Glu99 present at the surface of the protein (Fig. 4 ▸ *a*). A similar role of cadmium in crystal formation was observed for histidine-binding protein (Yao *et al.*, 1994 ▸), dipeptide-binding protein (Nickitenko *et al.*, 1995 ▸) and leucine-specific protein (Trakhanov & Quiocho, 1995 ▸).

The Cel5A initial protein crystallization solution contained 20 m*M* of three different salts: nickel(II) chloride (NiCl_2_), magnesium chloride (MgCl_2_) and cadmium chloride (CdCl_2_). When Ni^2+^ and Mg^2+^ were removed from the solution, the Cel5A protein sample still crystallized; however, when Cd^2+^ was removed, Cel5A no longer crystallized. Experiments using different concentrations of cadmium chloride were performed, and the protein could be crystallized in the presence of only 0.25 m*M* CdCl_2_ (80 times less than the original concentration and a 1:1 molar ratio between Cel5A and CdCl_2_). The small amount of CdCl_2_ (0.25 m*M*) also did not drastically change the ionic strength of the crystallization solution, but it was essential for success of crystallization experiments, indicating that Cd^2+^ was involved in the crystal packing. Moreover, the combination of Ni^1+^ and Cd^2+^ produced Cel5A crystals that belonged to the *P*1 space group. Using only Cd^2+^ produced Cel5A crystals that belonged to the *P*2_1_ space group with a completely different morphology (Fig. 4 ▸ *b*). The presence or absence of MgCl_2_ did not make any difference to Cel5A crystal formation. The Cel5A crystals demonstrate that manipulating the types of metals present in a crystallization solution could affect the way a protein packs in a crystal lattice. Different kinds of crystal packing (space groups) from a single target are always welcome from the crystallization experiment since they can have a strong influence on many of the factors that ultimately result in how well a crystal diffracts.

Cadmium has been identified in 864 PDB entries (∼0.7% of the 114 424 total entries), although it is possible that there are other structures where the ion has not been correctly identified. In light of the potentially important role in crystal formation, the Berkeley Screen has two entries (∼2%) containing CdCl_2_ to cover the possibility of some protein targets requiring Cd^2+^ for specific crystal contacts such as those seen in Cel5A crystals (Pereira *et al.*, 2010 ▸).

## 4. Small molecules function as bridges between symmetry mates (bis-tris propane buffer)   

**Figure 5:**
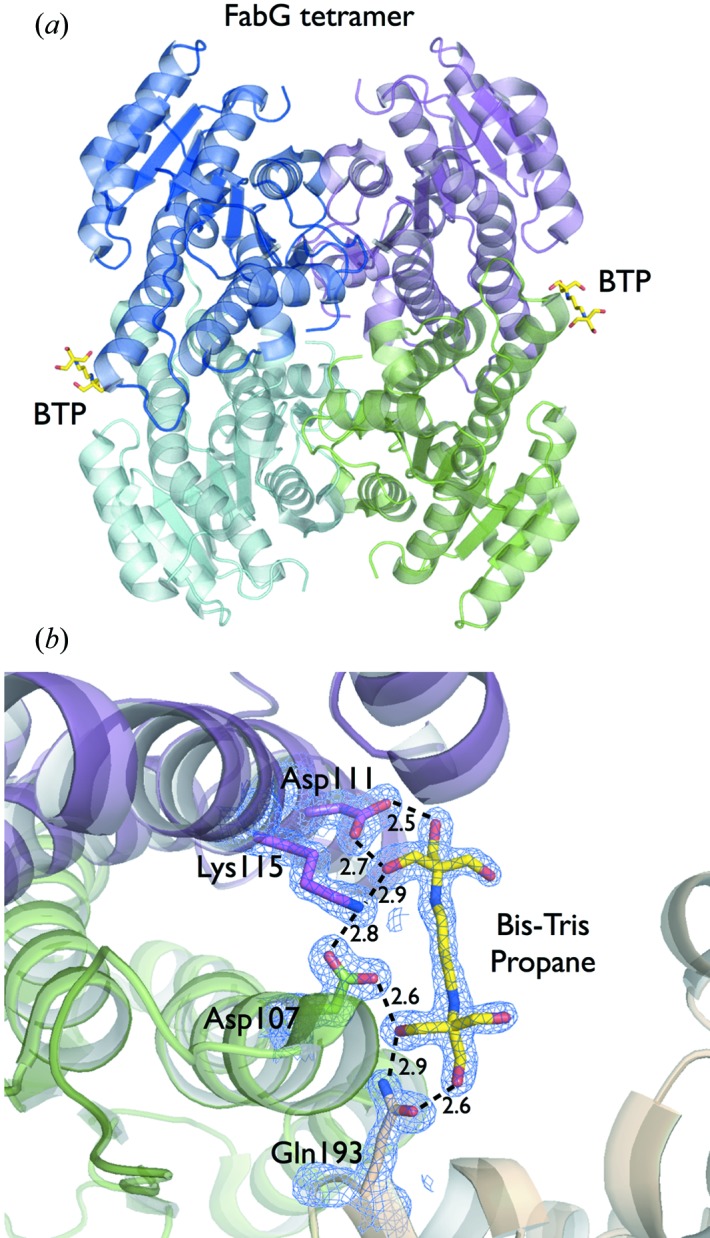
(*a*) The bis-tris propane molecules (BTP) bound between the two monomers on each side of the biological tetramer in the structure of FabG from *Cupriavidus taiwanensis* solved at 1.6 Å resolution (PDB code 4nbv; Javidpour *et al.*, 2014 ▸). (*b*) The crystal contact interface with a 2*mF*_o_–*DF*_c_ electron density map contoured at 1.2σ is shown in blue around Asp107, Asp111, Lys115, Gln193 and the bis-tris propane molecule. Bis-tris propane stabilizes the charged residues Asp111 and Lys115 in one monomer and Asp107 in the other monomer. The crystal contact is coordinated by bis-tris propane and Gln193 from the symmetry-related copy. Contacts are shown as broken lines and distances in ångströms.

The combination of bis-tris propane and citric acid is often used as a buffer in crystallization solutions. The bis-tris propane molecule can form crystal bridges between symmetry-related molecules, while citric acid, which is used to adjust the pH, can also compete for water solvent, forcing the protein out of the solution to form crystals. The structure of FabG from *Cupriavidus taiwanensis* (Javidpour *et al.*, 2014 ▸), solved at 1.6 Å resolution, is a good example of bis-tris propane in crystal packing. The FabG protein was crystallized from 0.1 *M* sodium malonate pH 7.0, 0.03 *M* citric acid, 0.07 *M* bis-tris propane pH 7.6 and 20% PEG 3350. Bis-tris propane molecules were bound between the two monomers on each side of the biological unit tetramer in the crystal (Fig. 5 ▸ *a*). The bis-tris propane stabilizes the charged residues Asp111 and Lys115 for one monomer and Asp107 for the other monomer. Finally, the crystal contact coordinates bis-tris propane and the symmetry mate chain using the Gln193 residue (Fig. 5 ▸ *b*). Adding bis-tris propane as one of the regular buffers used in the Berkeley Screen formulation exposes the crystallization targets to this small molecule involved in crystal lattice formation during initial crystallization trials. The Berkeley Screen has seven solutions containing bis-tris propane as a buffer.

## 5. Conclusion   

Initially, protein targets selected for X-ray crystallography studies depended on the availability of large quantities from natural sources. Advances in molecular biology methods have provided more freedom to macromolecular crystallographers in target selection and allowed great strides in elucidating fundamental biological questions using structural data. However, the amount of sample can often be a limiting factor for targets in modern protein X-ray crystallography; consequently the number of solutions chosen to be tested during crystallization must be done critically. Understanding the factors involved in crystallization is an essential step to selecting a good set of solutions, and the Berkeley Screen, despite limited release, has already been a valuable alternative to the commercially available screens, providing crystals for several publications in the past few years (Pereira *et al.*, 2017 ▸, 2016 ▸, 2014 ▸; Marcos *et al.*, 2017 ▸; Boyken *et al.*, 2016 ▸; Fallas *et al.*, 2016 ▸; Mills *et al.*, 2016 ▸; Eudes *et al.*, 2016 ▸; Helmich *et al.*, 2016 ▸; Javidpour *et al.*, 2014 ▸).

## 6. Additional Information   

An Excel file containing the screen formulation, individual chemical information such as the catalog number from Hampton Research for all the stock solutions, and the calculated volumes for each stock solution necessary to make 10 ml of each Berkeley Screen condition is provided as supporting information (supplementary file S2).

The group definition for the Berkeley Screen solution has been created by Art Robbins Instruments to be used on the Scorpion Screen Builder Robot. Requests for information about the Berkeley Screen group definition can be made directly to Art Robbins (http://www.artrobbins.com/contact-support).

## Supplementary Material

Supplementary File S1 - Berkeley Screen Formulation. DOI: 10.1107/S1600576717011347/ei5025sup1.pdf

Supplementary File S2 - An Excel file was created containing the screen formulation, individual chemical information such as the catalog number from Hampton Research for all the stock solutions, and the calculated volumes for each stock solution necessary to make 10 ml of each Berkeley Screen condition. DOI: 10.1107/S1600576717011347/ei5025sup2.xlsx

## Figures and Tables

**Table 1:** The distribution of the pH and the most common salts/precipitants among the BMCD entries and Berkeley Screen solutions

	BMCD (%)†	Berkeley Screen solutions (%)
Distribution of pH values		
pH range 3–4	2	4
pH range 4–5	10	11
pH range 5–6	19	19
pH range 6–7	28	24
pH range 7–8	29	27
pH range 8–9	10	13
pH range 9–10	2	2

Most common salts and precipitants		
PEG (all molecular weights)	43	73
Ammonium sulfate	26	12
Sodium chloride	14	10
Magnesium chloride	9	16
2-Methyl-2,4-pentanediol	9	9
2-Propanol	3	7
Lithium sulfate	3	6

† A total of 14 372 crystal entries were used to generate the data (Tung & Gallagher, 2009 ▸).
