# Supplementary material for: Berkeley Screen: a set of 96 solutions for general macromolecular crystallization
Source: J Appl Crystallogr. 2017 Sep 5;50(Pt 5):1352–8. doi: 10.1107/S1600576717011347 (PMC5627680; doi:10.1107/S1600576717011347)
Supplement: Supplementary file 1 [file j-50-01352-sup1.pdf]

Berkeley Screen Formulation

Solution	Salt	Solution	Buffer	Solution	Precipitant
A1.	0.05 M Calcium Chloride	A1.	0.1 M TRIS-HCl pH 8.0	A1.	2.2 M Sodium Chloride
A2.	None	A2.	0.1 M Sodium Acetate trihydrate pH 4.5	A2.	2.0 M Sodium Formate
A3.	None	A3.	0.1 M Sodium Citrate pH 5.3	A3.	2.0 M Ammonium Sulfate
A4.	None	A4.	0.1 M BIS-TRIS pH 6.5	A4.	1.8 M Ammonium Sulfate<br>10% 2-propanol
A5.	None	A5.	None	A5.	2.2 M Sodium malonate pH 5.0
A6.	None	A6.	None	A6.	2.2 M Sodium malonate pH 7.0
A7.	None	A7.	None	A7.	2.2 M Sodium malonate pH 8.0
A8.	0.2 M Potassium Chloride	A8.	0.1 M Hepes pH 7.5	A8.	30 % Pentaerythritol propoxylate (5/4 PO/OH)
A9.	0.1 M Magnesium Chloride	A9.	0.1 M Sodium acetate trihydrate pH 4.5	A9.	30 % Pentaerythritol propoxylate (5/4 PO/OH)
A10.	0.2 M Ammonium Acetate	A10.	0.1 M BIS-TRIS pH 6.5	A10.	25 % Pentaerythritol propoxylate (5/4 PO/OH)
A11.	0.4 M Sodium Chloride	A11.	0.1 M MES pH 5.5	A11.	25 % Pentaerythritol propoxylate (5/4 PO/OH)
A12.	None	A12.	0.1 M Hepes pH 7.5	A12.	35 % PEG 3,350<br>10 % 2-propanol
B1.	0.4 M Sodium Chloride	B1.	0.1M Tris-HCl pH 8.5	B1.	30% PEG 3,350
B2.	0.4 M Sodium Chloride	B2.	0.1M Hepes pH 7.5	B2.	30% PEG 3,350
B3.	0.4 M Sodium Chloride	B3.	0.1M BIS-TRIS pH 6.5	B3.	30% PEG 3,350
B4.	0.1 M Potassium - Sodium Tartrate<br>0.005 M Magnesium Chloride	B4.	None	B4.	25 % PEG 3,350
B5.	0.2 M Lithium Sulfate	B5.	None	B5.	20% PEG 3350
B6.	0.2 M Sodium Fluoride	B6.	None	B6.	20% PEG 3350
B7.	None	B7.	0.04 M Citric Acid, 0.06 M BIS-TRIS propane pH 6.4	B7.	25 % PEG 3,350
B8.	None	B8.	0.03 M Citric Acid, 0.07 M BIS-TRIS propane pH 7.6	B8.	25 % PEG 3,350
B9.	0.2 M Magnesium Chloride	B9.	0.1 M BIS-TRIS pH 6.5	B9.	25 % PEG 3,350
B10.	0.025 M Magnesium Chloride	B10.	0.1M TRIS pH 8.0	B10.	25% PEG 3350
B11.	0.2 M Ammonium Sulfate	B11.	0.1M Tris-HCl pH 8.5	B11.	25% PEG 3350
B12.	0.2 M Ammonium Sulfate	B12.	0.1M Hepes pH 7.5	B12.	25% PEG 3350
C1.	0.2 M Ammonium Sulfate	C1.	0.1M BIS-TRIS pH 6.5	C1.	25% PEG 3350
C2.	0.2 M Ammonium Sulfate	C2.	0.1 M MES pH 5.5	C2.	25% PEG 3350
C3.	0.1 M Calcium Chloride	C3.	None	C3.	20% PEG 3350
C4.	0.1 M Magnesium Chloride	C4.	0.1 M MES pH 5.5	C4.	20% PEG 3350
C5.	0.3 M Sodium Citrate	C5.	0.1 M Hepes pH 7.5	C5.	15 % PEG 3,350
C6.	0.2 M Potassium Chloride	C6.	None	C6.	25 % PEG 3,350
C7.	0.1 M di-Ammonium Phosphate	C7.	None	C7.	25 % PEG 3,350
C8.	0.1 M Sodium Chloride<br>0.1M Magnesium Chloride	C8.	None	C8.	20 % PEG 3,350

C9.	0.1 M Sodium Acetate	C9.	0.1 M BIS-TRIS pH 6.5	C9.	25 % PEG 3,350
C10.	0.1 M di-Ammonium Phosphate	C10.	0.1 M Sodium Acetate pH 4.5	C10.	20 % PEG 3,350<br>5% 2-propanol
C11.	0.1 M Sodium malonate pH 7.0	C11.	0.04 M Citric Acid, 0.06 M BIS-TRIS propane pH 6.4	C11.	20 % PEG 3,350
C12.	0.1 M Sodium malonate pH 7.0	C12.	0.03 M Citric Acid, 0.07 M BIS-TRIS propane pH 7.6	C12.	20 % PEG 3,350
D1.	0.1 M Sodium malonate pH 7.0	D1.	0.1 M TRIS-HCl pH 8.5	D1.	20 % PEG 3,350
D2.	0.2 M Potassium - Sodium Tartrate	D2.	0.1 M Hepes pH 7.5	D2.	20 % PEG 3,350
D3.	0.2 M Potassium - Sodium Tartrate	D3.	0.1 M BIS-TRIS pH 6.5	D3.	20 % PEG 3,350
D4.	0.2 M Sodium Acetate pH 4.5	D4.	None	D4.	25 % PEG 3,350
D5.	0.2 M Ammonium Acetate	D5.	0.1 M Hepes pH 7.5	D5.	25 %PEG 3,350
D6.	0.2 M Ammonium Acetate	D6.	0.1 M MES pH 5.5	D6.	25 %PEG 3,350
D7.	0.1 M Potassium Chloride<br>0.1 M di-Potassium Phosphate	D7.	None	D7.	30 % PEG 3,350
D8.	0.1 M di-Ammonium Citrate	D8.	0.1 M MES pH 5.5	D8.	20 % PEG 3,350<br>5% 2-propanol
D9.	0.1 M di-Ammonium Phosphate	D8.	0.1 M BIS-TRIS pH 6.5	D8.	20 % PEG 3,350<br>5% 2-propanol
D10.	0.1 M Lithium Sulfate<br>0.1 M Magnesium Chloride	D10.	0.1 M Hepes pH 7.5	D10.	20% PEG 3350<br>10 % hexanediol
D11.	0.1 M Lithium Sulfate<br>0.1 M Sodium Chloride	D11.	0.1 M BIS-TRIS pH 6.5	D11.	20% PEG 3350<br>10 % hexanediol
D12.	None	D12.	0.1 M Sodium Acetate pH 4.5	D12.	20 % PEG MME 2,000
E1.	0.2 M Sodium Chloride	E1.	None	E1.	25 % PEG MME 2,000
E2.	0.2 M Ammonium Sulfate	E2.	None	E2.	25 % PEG MME 2,000
E3.	0.6 M Magnesium Chloride	E3.	0.1 M MES pH 5.5	E3.	15 % PEG MME 2,000
E4.	1.5 M Ammonium sulfate	E4.	0.1 M Hepes pH 7.5	E4.	None
E5.	1.2 M Ammonium sulfate	E5.	0.1 M BIS-TRIS pH 6.5	E5.	None
E6.	0.2 M Sodium malonate pH 5.0	E6.	0.1 M Sodium Citrate pH 5.5	E6.	20 % PEG MME 2,000
E7.	0.2 M Sodium malonate pH 7.0	E7.	0.1 M BIS-TRIS pH 6.5	E7.	20 % PEG MME 2,000
E8.	0.2 M Sodium malonate pH 7.0	E8.	0.1 M Hepes pH 7.5	E8.	20 % PEG MME 2,000
E9.	0.2 M Sodium malonate pH 8.0	E9.	0.1 M TRIS-HCl pH 8.5	E9.	20 % PEG MME 2,000
E10.	0.2 M Sodium Chloride	E10.	None	E10.	20 % PEG MME 2,000<br>10 % Hexanediol
E11.	0.2 M di-Ammonium Phosphate	E11.	None	E11.	25 % PEG MME 550
E12.	0.2 M di-Sodium Tartrate	E12.	0.1 M Hepes pH 7,5	E12.	25 % PEG MME 550
F1.	0.8 M Ammonium sulfate	F1.	None	F1.	10 % PEG MME 550
F2.	0.2 M Magnesium Chloride	F2.	None	F2.	25 % PEG MME 550
F3.	0.2 M Ammonium acetate	F3.	None	F3.	20 % PEG MME 5,000
F4.	2.0 M Sodium Formate	F4.	0.1 M MES pH 5.5	F4.	5 % PEG MME 5,000
F5.	2.0 M Sodium Formate	F5.	0.1 M BIS-TRIS pH 6.5	F5.	5 % PEG MME 5,000
F6.	2.0 M Sodium Formate	F6.	0.1 M Hepes pH 7.5	F6.	5 % PEG MME 5,000

F7.	0.2 M Lithium sulfate	F7.	0.1 M TRIS-HCl pH 8.5	F7.	20 % PEG MME 5,000
F8.	None	F8.	0.1 M Hepes pH 7.5	F8.	55 % 2-methyl-2,4-pentanediol
F9.	None	F9.	0.1 M MES buffer pH 6.5	F9.	30% of 2-methyl-2,4-pentanediol
F10.	0.01 M Magnesium Chloride	F10.	0.05 M Hepes pH 7.2<br>0.05 M Sodium Citrate pH 5.8	F10.	12% 2-methyl-2,4-pentanediol
F11.	0.1 M Sodium Acetate	F11.	0.1 M MES pH 5.5	F11.	40 % 2-methyl-2,4-pentanediol
F12.	0.1 M Sodium Formate	F12.	0.1 M Sodium Citrate pH 5.5	F12.	35 % 2-methyl-2,4-pentanediol
G1.	0.4 M Lithium Sulfate	G1.	0.1 M TRIS-HCl pH 8.0	G1.	25 % 2-methyl-2,4-pentanediol
G2.	0.2 M Sodium Malonate pH 7.0	G2.	0.1 M BIS-TRIS pH 6.5	G2.	45 % 2-methyl-2,4-pentanediol
G3.	0.1 M Sodium citrate	G3.	0.1 M Hepes pH 7.5	G3.	30 % 2-methyl-2,4-pentanediol
G4.	0.4 M Magnesium chloride	G4.	0.1 M Sodium Acetate pH 4.5	G4.	20 % 2-methyl-2,4-pentanediol
G5.	0.1 M Potassium Chloride	G5.	0.04 M Citric Acid, 0.06 M BIS-TRIS propane pH 6.4	G5.	15 % 2-methyl-2,4-pentanediol
G6.	0.5 M di-Ammonium Tartrate	G6.	None	G6.	10 % 2-methyl-2,4-pentanediol
G7.	1.5 M Ammonium Sulfate	G7.	0.1 M Sodium acetate pH 4.5	G7.	5 % 2-methyl-2,4-pentanediol
G8.	0.8 M Ammonium Phosphate	G8.	0.1 M BIS-TRIS pH 6.5	G8.	15 % 2-methyl-2,4-pentanediol
G9.	None	G9.	0.1 M BIS-TRIS pH 6.5	G9.	45 % PEG 400
G10.	0.2 M Magnesium Chloride	G10.	0.1 M Hepes pH 7.5	G10.	30% PEG 400
G11.	0.6 M Magnesium Chloride	G11.	0.1 M Hepes pH 7.5	G11.	25% PEG 400
G12.	0.02 M Cadmium Chloride	G12.	0.025 M Citric Acid, 0.075 M BIS-TRIS propane pH 8.2	G12.	25 % PEG 400
H1.	0.2 M di-Sodium Tartrate	H1.	0.1 M Sodium Acetate pH 4.5	H1.	25 % PEG 400
H2.	0.2 M Sodium Fluoride	H2.	0.1 M MES pH 5.5	H2.	20 % PEG 400
H3.	0.1 M Sodium Chloride<br>0.2 M Magnesium Chloride	H3.	None	H3.	25 % PEG 400
H4.	0.1 M Sodium Acetate	H4.	0.04 M Citric Acid, 0.06 M BIS-TRIS propane pH 6.4	H4.	25 % PEG 400
H5.	0.1 M Lithium sulfate<br>0.1 M Magnesium Chloride	H5.	None	H5.	25 % PEG 400
H6.	0.1 M Calcium Chloride	H6.	0.1 M MES pH 5.5	H6.	20 % PEG 1,500<br>12 % Hexanediol
H7.	0.1 M Cadmium Chloride	H7.	0.1 M Hepes pH 7.5	H7.	20 % PEG 1,500<br>5 % 2-propanol
H8.	0.2 M Ammonium Acetate	H8.	0.1 M Sodium Acetate pH 4.6	H8.	30 % PEG 4,000
H9.	None	H9.	0.1 M Hepes pH 7.5	H9.	25 % PEG 4,000<br>10 % 2-propanol
H10.	0.2 M Sodium Acetate	H10.	0.1 M BIS-TRIS pH 6.5	H10.	25 % PEG 4,000
H11.	0.1 M Magnesium Chloride<br>0.1 M Calcium Chloride	H11.	None	H11.	20 % PEG 8,000, 10 % Hexanediol
H12.	0.2 M Ammonium Acetate	H12.	0.1 M TRIS-HCl pH 8.0	H12.	15 % PEG 10,000
